# What Accounts for the Increased Incidence of Gynecomastia Diagnosis in Denmark from 1998–2017?

**DOI:** 10.1210/clinem/dgaa485

**Published:** 2020-07-25

**Authors:** Glenn D Braunstein

**Affiliations:** Department of Medicine, Cedars-Sinai Medical Center, Los Angeles, California

**Keywords:** gynecomastia, breast hypertrophy, pseudogynecomastia

Gynecomastia is the benign proliferation of breast glandular tissue in males (1). This definition differentiates it from breast enlargement due to the deposition of fat (pseudogynecomastia, lipomastia), neoplasms, or inflammation. All males have some breast glandular tissue; gynecomastia often represents an imbalance between the stimulatory effects of estrogen and the inhibitory effects of androgens on the glandular component, while pseudogynecomastia is commonly found in boys and men who are overweight (1, 2). Nevertheless, some authors and physicians lump all causes of breast enlargement in males together under the term “gynecomastia” (3). This situation is not helped by the diagnostic terminology utilized by the tenth revision of the International Statistical Classification of Diseases and Related Health Problems, which lists “gynecomastia” under Code N62, which also includes hypertrophy of the breast from any benign cause as well as true gynecomastia.

The prevalence of gynecomastia has been studied in different populations and 3 major peaks over the lifespan have been found: infants (60–90% with gynecomastia), pubertal and young adults (4–69% with gynecomastia), and aging males (24–65% with gynecomastia) (1). The wide range of prevalence is due, in part, to differences in the definition of gynecomastia. In some studies, gynecomastia was diagnosed if the breast tissue diameter was ≥ 0.5 cm by palpation, while in others, the minimum diameter of breast glandular tissue required was ≥ 2 cm. Recently, Klang and co-workers examined quantitative CT-scans performed on males from the general population who were being evaluated for trauma (4). Breast tissue diameter ranged from 0.1–5.6 cm, with a mean of 1.24 cm. The 90^th^ percentile was 2.2 cm, the 95^th^ percentile was 2.8 cm, and the 97.5^th^ percentile was 3.6 cm (4). Based upon this objective data and the size cutoff advocated by experienced clinicians, a diagnosis of gynecomastia should require that the breast glandular tissue be ≥ 2 cm (4, 5). The utilization of this value in future studies should decrease some of the variability in its reported prevalence.

Despite an abundance of prevalence studies, the report by Koch and co-workers in this journal provides the first incidence data for gynecomastia (6). Koch et al queried the Danish National Patient Registry, which contains information from inpatient and outpatient public and private hospital diagnoses and treatment for the entire Danish population, which in 2019 included 2.89 million men. The ability to capture medical information on the whole population decreased the potential for selection bias. As noted above, the ICD-10 codes used in most countries for gynecomastia are not specific for gynecomastia. In contrast, Denmark uses extensions that allow a specific diagnosis of gynecomastia (code N62.9A), excluding other causes of breast enlargement. This combination of complete or near-complete capture of a new gynecomastia diagnosis for the entire male population over a 20-year period (1998–2017) provided the authors with an invaluable resource to look at the incidence of new gynecomastia diagnoses as well as the trend over time. They showed that the average 20-year incidence rate was 3.4 per 10 000 men per year, with the largest averages being for those in the 16–20- and 61–80-year-old groups (6.5 and 4.6/10 000/year, respectively). Over the 20 years, they found a 5-fold increase in incidence in the 16–20-year-old group, and a greater than 10-fold increase in the 10–15-, 21–40-, 41–60-, and 61–80-year-old groups. Most of the increase in incidence occurred in the first 15 years, from 1998–2012, with a lower rate of rise being found from 2011–2017. Two broad peaks of age-specific incidence were noted: pubertal/young adults (14–30 years old) and the elderly (70–80 years old), confirming the qualitative pattern found with the prior prevalence studies. The authors speculate that the increasing incidence over time could be due to changes in the sex steroid hormone environment, possibly due to environmental endocrine-disrupting chemicals or the increasing incidence of obesity, androgen abuse, use of medications that have been associated with gynecomastia, and/or changes in the prevalence of diseases associated with gynecomastia, such as testicular tumors.

Although this study has many strengths, it also has several weaknesses, which the authors clearly acknowledge. It is a retrospective registry study that depends on the accuracy of the diagnosis. Because of the type of approval they received from the Danish Data Protection Agency, the authors were not able to examine patient charts to determine how the patient was examined and whether there was any quantification of the size of the breast glandular tissue. They were also not able to determine how the patients were ascertained. Did patients seek out physicians because of breast enlargement, pain, tenderness, or embarrassment, or was the gynecomastia an incidental finding on a clinical examination? Does the increase in incidence over the 20 years represent a real increase in gynecomastia or an ascertainment bias? For instance, Klang and co-workers indicate that “the increased usage of CT scans results in incidental finding of gynecomastia…[and] patients are frequently referred for endocrinological evaluation for this incidental finding” (4). In this regard, the number of CT scans performed in Denmark doubled from 2008–2015 (7), raising the possibility that at least some of the increase in gynecomastia diagnoses was due to finding incidental gynecomastia on a CT-scan performed for other reasons. Also, from 1998–2016, Danish males over the age of 18 exhibited a 16.3% and 59.3% increase in the prevalence of overweight and obesity, respectively (8). Thus, it is quite possible that some of the increased incidence of breast enlargement during those years was due to pseudogynecomastia misclassified as gynecomastia. In addition, the authors were not able to examine the database for some of the diseases and medications known to be associated with gynecomastia in order to address the question as to why the incidence of gynecomastia increased in Denmark over the 20-year period.

Despite these limitations, this is a very important study, which, hopefully, will stimulate these investigators to seek permission to examine the health records (even in registry form) in greater detail and perform a multivariate analysis for possible associations that could explain the rising time trends in gynecomastia incidence. Also, other investigators in other countries should examine this same question to determine if the incidence of gynecomastia is rising worldwide.

Finally, the actual incidence of gynecomastia reported in this study is rather low considering the high prevalence of gynecomastia noted in adolescents and the elderly in many studies (1). This suggests that the apparent low incidence represents gross underreporting due to physicians not routinely examining the breasts in males unless the patient raises a concern. Hopefully, the studies of Koch et al (6) and Klang and colleagues (4) will stimulate health care providers to routinely examine the male breast and record their findings so that we may develop a more accurate assessment of the true incidence of gynecomastia.

## Data Availability

Data sharing is not applicable to this article, as no datasets were generated or analyzed during the current study.

## Additional Information

***Disclosure Summary:*** Dr Braunstein is a defense expert witness for Janssen/Johnson & Johnson for their risperidone and gynecomastia litigation.
